# A systematic review on the prevalence of symptoms of depression, anxiety and distress in long‐term cancer survivors: Implications for primary care

**DOI:** 10.1111/ecc.13086

**Published:** 2019-05-14

**Authors:** Daan Brandenbarg, Saskia W. M. C. Maass, Olaf P. Geerse, Mariken E. Stegmann, Charlotte Handberg, Maya J. Schroevers, Saskia F. A. Duijts

**Affiliations:** ^1^ Department of General Practice and Elderly Care Medicine, University Medical Center Groningen University of Groningen Groningen The Netherlands; ^2^ Department of Pulmonary Diseases and Tuberculosis, University Medical Center Groningen University of Groningen Groningen The Netherlands; ^3^ Department of Public Health, Faculty of Health Aarhus University Aarhus Denmark; ^4^ The Danish National Rehabilitation Center for Neuromuscular Diseases Aarhus Denmark; ^5^ Department of Health Psychology, University Medical Center Groningen University of Groningen Groningen The Netherlands; ^6^ Department of Public and Occupational Health, Amsterdam Public Health Research Institute, Amsterdam UMC Vrije Universiteit Amsterdam Amsterdam The Netherlands

**Keywords:** anxiety, cancer survivors, depression, distress, long term, psychological problems

## Abstract

**Introduction:**

Symptoms of depression, anxiety and distress are common in the first years after a cancer diagnosis, but little is known about the prevalence of these symptoms at the long term. The aim of this review was to describe the prevalence of symptoms of depression, anxiety and distress in long‐term cancer survivors, five or more years after diagnosis, and to provide implications for primary care.

**Methods:**

We performed a systematic literature search in the PubMed, PsycINFO and CINAHL databases. Studies were eligible when reporting on the prevalence of symptoms of depression, anxiety and/or distress in long‐term cancer survivors (≥5 years after diagnosis), treated with curative intent.

**Results:**

A total of 20 studies were included. The reported prevalence of depressive symptoms (*N* = 18) varied from 5.4% to 49.0% (pooled prevalence: 21.0%). For anxiety (*N* = 7), the prevalence ranged from 3.4% to 43.0% (pooled prevalence: 21.0%). For distress (*N* = 4), the prevalence ranged from 4.3% to 11.6% (pooled prevalence: 7.0%).

**Conclusion:**

Prevalences of symptoms of depression, anxiety and distress among long‐term survivors of cancer do not fundamentally differ from the general population. This is reassuring for primary care physicians, as they frequently act as the primary physician for long‐term survivors whose follow‐up schedules in the hospital have been completed.

## 1 INTRODUCTION

In Europe, there were an estimated 3.9 million new cases of cancer in 2018 (Ferlay et al., 2018). In general, survival of most types of cancer has increased over the last decade. Five‐year cancer survival rates in 2017 in the Netherlands and the United States were 64% and 67% respectively (Netherlands Cancer Registry, 2019; Siegel, Miller, & Jemal, 2019). It is expected that in 2020, there will be roughly 660,000 patients with cancer in the Netherlands, a fair proportion of which being a cancer survivor (Dutch Cancer Society, 2011). Survivors may face various physical and psychosocial sequelae entailed by their illness or treatment. These sequelae can be severe, debilitating and sometimes even permanent. Frequently reported symptoms are fatigue, cognitive impairment, sexual dysfunction, depression, anxiety and distress (Bloom, 2002; Deimling, Bowman, Sterns, Wagner, & Kahana, 2006; Yi & Syrjala, 2017). Yet, psychological problems, such as depression and anxiety, are often under‐diagnosed and under‐treated among patients with cancer, and need early identification (Walker, 2014).

Until now, most studies focusing on psychological problems in cancer survivors have explored their prevalence mainly within the first years after diagnosis, when patients are still in follow‐up at the hospital (Stanton, 2006). For example, Krebber et al. (2014) found, in their meta‐analysis, a pooled prevalence of depressive disorder among cancer patients of 14% during treatment, 9% in the first year post‐treatment and 8% one year or more post‐treatment (Krebber et al., 2014). Moreover, Watts, Prescott, Mason, McLeod, and Lewith (2015) reported in their systematic review pre‐treatment, on‐treatment and post‐treatment anxiety prevalence rates in ovarian cancer patients to be 19%, 26% and 27% respectively (Watts et al., 2015). Conversely, long‐term prevalence of (symptoms of) depression, anxiety and/or distress beyond the first 5 years, in cancer survivors with a range of tumour types, has received limited attention so far. Nevertheless, some specific tumour types have been studied already, for example, Maass, Roorda, Berendsen, Verhaak, and Bock (2015) reported a higher prevalence of symptoms of depression among long‐term (>2 years) breast cancer survivors, compared to the general female population (Maass et al., 2015). However, none of the reviews conducted so far focused solely on survivors of cancer at least 5 years after diagnosis.

Focusing on the period beyond the first years after diagnosis is important, since most cancer follow‐up schedules in hospitals discontinue after 5 years. This 5‐year mark is consistent with the view commonly held by the general population, that those who have survived for 5 years have a relatively high probability of “being cured” (Deimling, 2007). In countries such as the Netherlands, in which the general practitioners (GPs) act as gatekeepers in the healthcare system, the GP typically is the primary physician for long‐term cancer survivors after the follow‐up period. For these GPs, it is important to know how prevalent psychological symptoms are among long‐term cancer survivors, in order to be able to provide appropriate or proactive care.

Thus, the aim of this systematic review was to describe the prevalence of symptoms of depression, anxiety and/or distress in long‐term cancer survivors, five or more years after diagnosis, and to provide an overview of implications, related to these psychological symptoms in long‐term cancer survivors, for primary care.

## 2 METHODS

### 2.1 Protocol registration and report

The protocol of this review is available at PROSPERO, the international database of prospectively registered systematic reviews for health and social care (registration number CRD42018110822). The Preferred Reporting Items for Systematic Reviews (PRISMA statement) was used as a formal guideline for systematic reviews (Moher, Liberati, Tetzlaff, Altman, & PRISMA Group, 2010).

### 2.2 Search strategy

A systematic search for publications in English has been conducted in the electronic databases PubMed (MEDLINE), PsycINFO (Ovid) and CINAHL (EBSCO), from 2000 to September 30, 2018. Studies were identified using a search syntax based on the PubMed strategy, which uses a combination of MeSH terms and free text terms, and included synonyms of terms related to cancer, survivor, long term, depression, anxiety, distress and prevalence. Where necessary, the syntax was adapted for use in the other databases. The PubMed search syntax can be found in the Appendix.

### 2.3 Study selection

**Figure 1:**
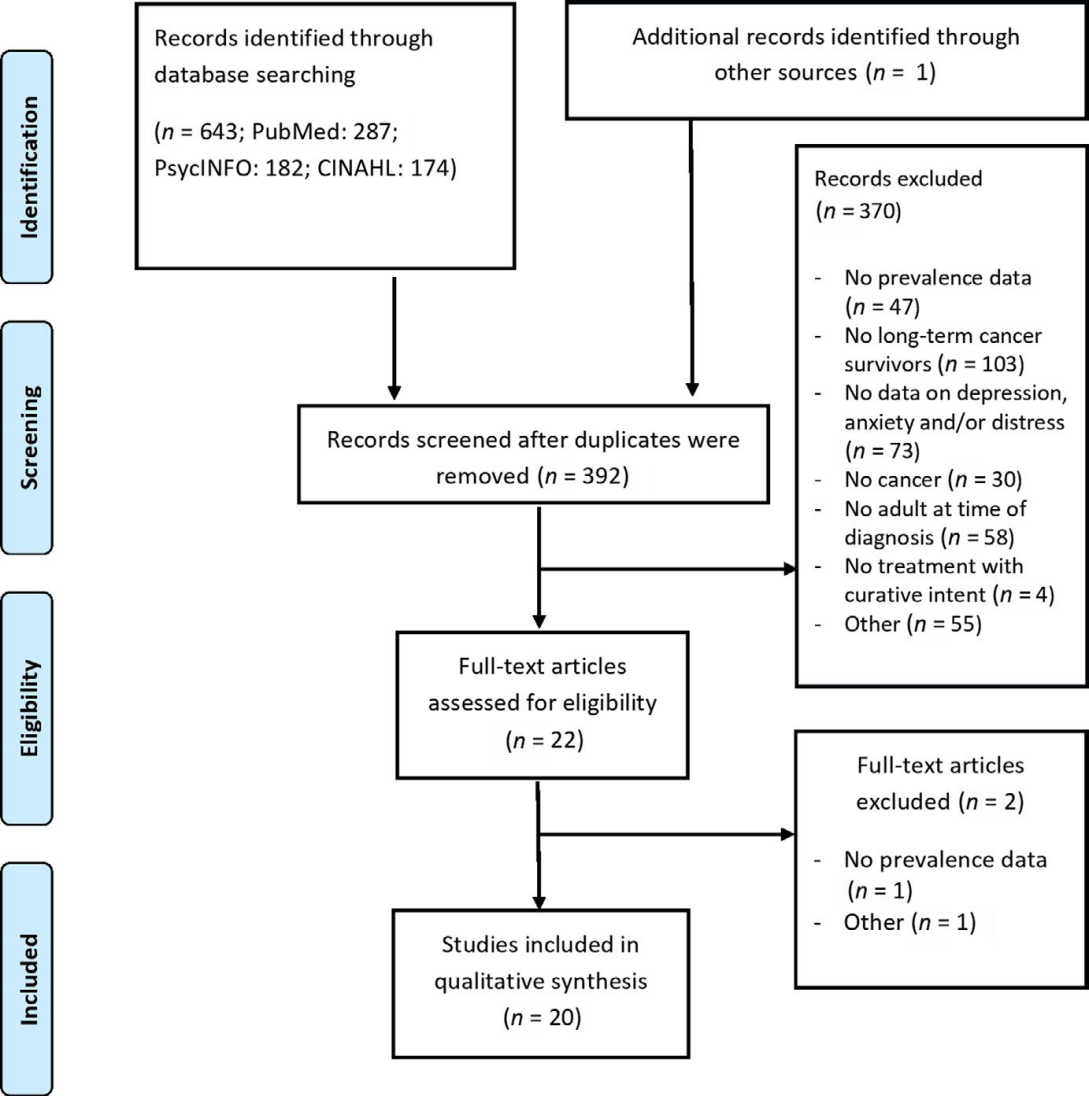
Flow diagram

To assess whether identified studies met the selection criteria, they were independently screened on title and abstract by two authors (SFAD and MJS). Full‐text articles were retrieved when there was not sufficient information to establish appropriateness for inclusion. A manual search of reference lists of selected articles and relevant systematic reviews has been performed to identify further relevant studies. Studies were excluded for the following reasons: (a) no prevalence data; (b) no long‐term cancer survivors (≥5 years after diagnosis); (c) no data on symptoms of depression, anxiety and/or distress; (d) no cancer; (e) no adult (≥18 years old) at time of diagnosis; (f) no treatment with curative intent; and/or (g) other (e.g., full text not available, design paper) (Figure 1). In case of disagreement during the selection process, a third author (DB) decided upon the eligibility of a study.

### 2.4 Data extraction and synthesis

A data extraction form was developed to record relevant study details. Data were independently extracted by two authors (OPG and MES) and included the following: (a) general study characteristics (e.g., author, year of publication, country), (b) study characteristics (e.g., recruitment period, design, setting), (c) participant characteristics (e.g., age, gender, number, tumour type, time since diagnosis, treatment) and (d) symptom characteristics (e.g., measurement, prevalence [of symptoms of depression, anxiety and/or distress]). We defined symptoms of anxiety, depression and distress as follows: these have to be measured using a generic questionnaire or subscale of a questionnaire to assess symptoms of depression, anxiety or distress. The latter, distress, is typically defined by the presence of an adjustment disorder with or without depression/anxiety. All data were synthesised by describing the prevalence of symptoms of depression, anxiety and/or distress in adults with cancer assessed at least 5 years after diagnosis. Also, pooled point prevalences and 95% confidence intervals (CIs) were produced in STATA (version StataSE 15) and presented in forest plots. For this, we used the cut‐off values of >16 for the CES‐D, >8 for HADS‐A and HADS‐D, and values suggestive of possible depression, anxiety or distress for the other questionnaires.

### 2.5 Quality assessment

The methodological quality of the included studies was scored independently by two authors (DB and CH), by applying the 14 items of the NIH's quality assessment tool for Observational Cohort and Cross‐Sectional Studies (National Heart, Lung, & Blood Institute, 2014). We slightly adapted the summary score to be applicable for our review question. That is, we scored studies as “excellent” if they scored “Yes” on the following four questions: (a) Was the study population clearly specified and defined?; (b) Was the participation rate of eligible persons at least 50%?; (c) Were the exposure measures (independent variables) clearly defined, valid, reliable, and implemented consistently across all study participants?; and (d) Were the outcome measures (dependent variables) clearly defined, valid, reliable, and implemented consistently across all study participants? Exposure was defined as a diagnosis of cancer; outcome was defined as symptoms of depression, anxiety or distress. Studies received the result “excellent” if they scored positive on all four questions, “good” if they scored positive on three out of these four questions, “fair” if they scored positive on two questions and “poor” in case of less than two positive questions. In case of disagreement, items were discussed until consensus was reached, or if necessary, a third author (SFAD) was consulted.

## 3 RESULTS

### 3.1 Search results and characteristics of included studies

Our original search yielded 644 titles, 392 of which remained after the removal of duplicates. Of these titles, 22 met the criteria for a full‐text review, of which 20 studies were subsequently included (Figure 1). Agreement between researchers was 95.7% for title/abstract screening, and full consensus was reached after discussion. The third author was consulted in four cases during full‐text selection. Two studies were based on the same data set, but applied different inclusion criteria, resulting in two individual samples (Greenwald & McCorkle, 2008; McCorkle, Tang, Greenwald, Holcombe, & Lavery, 2006). A total of 14 studies had a cross‐sectional design (Boyes, Girgis, Zucca, & Lecathelinais, 2009; Chongpison et al., 2016; Dahl et al., 2005; Goo, Song, Shin, & Ko, 2016; Greenwald & McCorkle, 2008; Harrison et al., 2011; Henningsohn et al., 2003; Hoffman, McCarthy, Recklitis, & Ng, 2009; McCorkle et al., 2006; Pedersen, Rossen, Olesen, von der Maase, & Vedsted, 2012; Reyes‐Gibby, Anderson, Morrow, Shete, & Hassan, 2012; Schootman, Deshpande, Pruitt, Aft, & Jeffe, 2010; Sharpley et al., 2017; Vogel et al., 2017), and six were prospective cohort studies (Brunault et al., 2013; Chambers et al., 2012; Chen et al., 2013; Crespi, Ganz, Petersen, Castillo, & Caan, 2008; Funk, Karnell, & Christensen, 2012; Johansson et al., 2011). Most of the included studies were conducted in the United States (*N* = 10) (Chen et al., 2013; Chongpison et al., 2016; Crespi et al., 2008; Funk et al., 2012; Greenwald & McCorkle, 2008; Hoffman et al., 2009; McCorkle et al., 2006; Reyes‐Gibby et al., 2012; Schootman et al., 2010; Vogel et al., 2017); other studies were conducted in Europe (*N* = 6) (Brunault et al., 2013; Dahl et al., 2005; Henningsohn et al., 2003; Johansson et al., 2011; Pedersen et al., 2012), Australia/New Zealand (*N* = 3) (Boyes et al., 2009; Chambers et al., 2012; Sharpley et al., 2017) or Asia (*N* = 1) (Goo et al., 2016). In total, 17,726 patients (aged 21–93 years) were included across all studies. The majority of patients were diagnosed with breast, prostate or colorectal cancer. Most patients were surgically treated for their cancer, and a minority received radiotherapy or chemotherapy. The average time since diagnosis across studies, reporting on the median or mean number of months since treatment (*N* = 16), was 9.6 years (range 5–21 years). Further details on individual study and patients’ characteristics have been provided in Table 1.

**Table 1:** Study and patient characteristics

Author, year	Country	Study design	Study population	Age^a^	Gender; % female	Time since diagnosis/treatment	Cancer treatment^a^
Boyes et al. (2009)	Australia	Cross‐sectional	*N* = 863 Breast 29% Melanoma 15% Prostate 15%; Colorectal 13% Other 28%	Median 63 years Range 26−76 years	55%	Time since diagnosis: Range 5−6 years Mean 5.5 years; *SD* 3.0 months	Surgery 85% Radiotherapy 45% Chemotherapy 25% Hormone therapy 24%
Brunault (2013)	France	Prospective cohort	*N* = 120 Breast 100%	Mean 58.3 years *SD* 8.2 years	100%	Time since the end of treatment: Mean 8.1 years; *SD* 1.3 year Range 6.1–11.0 years	Surgery 100% Concurrent chemo/radiotherapy 51% Sequential chemo/radiotherapy 49% Hormonal therapy 46%
Chambers (2012)	Australia	Prospective cohort	*N* = 742 Colon 60% Rectal 31% Other 9%	20−49 years 7.0% 50−59 years 20.2% 60−69 years 36.4% 70−80 years 36.4%	46%	Time since diagnosis: 60 months	NR
Chen et al. (2013)	USA	Prospective cohort	*N* = 54 Head and neck 100%	T0 (*N* = 211) Median 57 years Range 21−93 years	42%	Time since completion of radiotherapy: 5 years	T0 (*N* = 211) Definitive radiotherapy 55% Post‐operative radiotherapy 45% Concurrent chemotherapy 42%
Chongpison (2016)	USA	Cross‐sectional	*N* = 576 Rectal 100%	Mean 72.8 years *SD* 10.9 years	41%	Time since diagnosis: Median 11.7 years Minimum 5 years Mean 13.0 years; *SD* 6.2 years	Anastomosis 57% Permanent ostomy 32% Temporary ostomy followed by anastomosis 11%
Crespi et al. (2008)	USA	Prospective cohort	*N* = 2,280 Breast 100%	Mean 66.3 years *SD* 10.1 years Range 34−89 years	100%	Time since diagnosis: Mean 7.4 years; *SD* 0.9 year Range 5.3–9.9 years	Conserving surgery 52% Mastectomy 48% Chemotherapy 57% Tamoxifen/aromatase inhibitor current 29% Tamoxifen/aromatase inhibitor past 50%
Dahl et al. (2005)	Norway	Cross‐sectional	*N* = 1,438 Seminoma testicular 50% Non‐seminoma testicular 50%	Mean 44.6 years *SD* 10.2 years	0%	Time since diagnosis: Mean 11.3 years; *SD* 4.2 years Range 5−21 years	Surveillance 8% Retroperitoneal lymph node dissection 11% Radiotherapy 43% Chemotherapy 39%
Funk et al. (2012)	USA	Prospective cohort	*N* = 337 Oral cavity 37.7% Oropharynx 22.3% Hypopharynx 7% Larynx 21.7% Other 16.3%	Median 57 years	NR	NR Inclusion: survived at least 5 years	Surgery only 39% Radiotherapy only 13% Surgery and radiotherapy 37% (= with or without chemo) Radiation and chemotherapy 11% Unknown 1%
Goo et al. (2016)	Korea	Cross‐sectional	*N* = 702 Stomach 61% Lung 15% Breast 6% Colorectal 6% Thyroid 4% Other 9%	Non‐depressive group: Mean 62.0 years; *SD* 10.4 years Depressive group: Mean 60.5 years; *SD* 11.4 years	44%	Time since treatment: Non‐depressive group: Mean 8.1 years; *SD* 3.2 years Depressive group: Mean 8.0 years; *SD* 2.2 years	Surgery 98% Chemotherapy 36% Radiotherapy 25%
Greenwald and McCorkle (2008)	USA	Cross‐sectional	*N* = 179 Cervical 100%	Mean 51.7 years; *SD* 8.7 years Mean age at diagnosis 37.8 years	100%	NR Minimum 6 years	Surgery 89% Hysterectomy 75% Ovary removal 35% Radiotherapy 21% Hormonal therapy 43%
Harrison et al. (2011)	UK	Cross‐sectional	*N* = 659 Breast 39.2% Colorectal 31.1% Prostate 29.7%	Mean 71.6 years; *SD* 9.9 years Range 42−92 years	54%	NR At least 5 years from diagnosis	Surgery 77% Radiotherapy 48% Chemotherapy 21% Hormonal therapy 35% Other treatments 3%
Henningsohn (2003)	Sweden	Cross‐sectional	*N* = 350 Bladder 100%	Men: Mean 71.0 years *SEM* 0.5 years Median 72 years Women: Mean 71.1 years *SEM* 1.2 years Median 74 years	NR	NR Results stratified for years after surgery (2−5 years/6−10 years/>11 years)	Surgery 100%
Hoffman et al. (2011)	USA	Cross‐sectional	*N* = 4,636 Female genital organs 25.2% Breast 22.9% Prostate/testes 13.9% Colorectal 9.6% Head/neck/lung 4.6% Leukaemia/lymphoma 5.9% Melanoma 9.6% Bladder/kidney 5.6% Other 14.2%	Median 66 years	65%	Time since diagnosis: Median 12 years	NR
Johansson et al. (2014)	Sweden/ Finland	Prospective cohort	*N* = 394 Prostate 100%	Median 77.0 years Range 61−88y	0%	Time since diagnosis: Median 12.2 years Range 7−17 years	Radical prostatectomy 50% Watchful waiting 50%
McCorkle et al. (2013)	USA	Cross‐sectional	*N* = 208 Cervical 100%	Median 54 years Range 29−92 years Mean 55.2 years *SD* 11.9 years	100%	Time since diagnosis: Mean 13.9 years Median 13 years	Surgery 86% Radiotherapy 30%
Pedersen et al. (2019)	Denmark	Cross‐sectional	*N* = 316 Testicular 100%	Mean 47.6 years *SD* 10.9 years	0%	Time since diagnosis: Mean 12.0 years *SD* 3.0 years	Orchidectomy 100% Chemotherapy 29%
Reyes‐Gibby et al. (2012)	USA	Cross‐sectional	*N* = 240 Breast 100%	Mean 58 years *SD* 16 years	100%	Time since treatment: Range 6−13 years Mean 7.9 years Median 8 years	Modified radical mastectomy 51% Segmental 27% Segmental sentinel biopsy 14% Total mastectomy 11% Radiotherapy 60%
Schootman et al. (2010)	USA	Cross‐sectional	*N* = 2,762 Bladder 2.2% Breast 21.1% Cervix 10.8% Colorectal 7.2% Lung 1.9% Lymphoma 2.8% Melanoma 7.1% Ovarian 3.4% Prostate 12.2% Thyroid 2.4% Uterus 7.0% Other 21.8%	NR White race (*N* = 2,380; 86%) 18−39y 9.6% 40−64 years 40.4% 65−84 years 43.8% 85+ years 6.2%	64%	Time since diagnosis: Mean 15.8 years 95% CI 15.2–16.3	NR
Sharpley et al. (2017)	Australia/New Zealand	Cross‐sectional	*N* = 146 Prostate 100%	Mean 77.0 years *SD* 6.8 years Range 57−92 years	0%	Time since treatment: 10 years	Hormonal therapy and radiotherapy 100%
Vogel et al. (2017)	USA	Cross‐sectional	*N* = 724 Melanoma 100%	Range 30−72 years 30−39 6.8% 40−49 16.2% 50−59 36.3% 60−72 40.8%	60%	Time since diagnosis: Mean 9.6 years *SD* 1.0 year	Surgery only 86% Lymph node dissection 35%

Abbreviations: *N*, number; NR, not reported; *SD*, standard deviation; *SEM*, standard error of the mean.

a Age/cancer treatment at time of measurement of prevalence of depression, anxiety and/or distress, unless stated otherwise.

### 3.2 Prevalence of symptoms of depression, anxiety and distress

Most studies detailed on the prevalence of depressive symptoms (*N* = 18, with data available for 8,803 patients) (Boyes et al., 2009; Brunault et al., 2013; Chambers et al., 2012; Chen et al., 2013; Chongpison et al., 2016; Crespi et al., 2008; Dahl et al., 2005; Funk et al., 2012; Goo et al., 2016; Greenwald & McCorkle, 2008; Harrison et al., 2011; Henningsohn et al., 2003; Johansson et al., 2011; McCorkle et al., 2006; Pedersen et al., 2012; Reyes‐Gibby et al., 2012; Sharpley et al., 2017; Vogel et al., 2017; Table 2). The prevalence of anxiety symptoms (*N* = 7; 4,855 patients) (Boyes et al., 2009; Chambers et al., 2012; Dahl et al., 2005; Harrison et al., 2011; Henningsohn et al., 2003; Johansson et al., 2011; Vogel et al., 2017) and distress symptoms (*N* = 4; 9,548 patients; Chambers et al., 2012; Dahl et al., 2005; Hoffman et al., 2009; Schootman et al., 2010) was reported less frequently (Table 2).

**Table 2:** Prevalence of symptoms of anxiety, depression and distress

Author, year	Measurement symptoms of depression and cut‐off	Prevalence symptoms of depression + *N* a	Measurement symptoms of anxiety and cut‐off	Prevalence symptoms of anxiety + *N* a	Measurement symptoms of distress and cut‐off	Prevalence symptoms of distress + *N* a
Boyes et al. (2009)	HADS‐D (range 0–21) Borderline 8–10 Clinical 11–21	*N* = 847^b^ 7.0% 4.0%	HADS‐A (range 0–21) Borderline 8–10 Clinical 11–21	*N* = 846 12.0% 9.0%	–	–
	HADS‐D (range 0–21) Median (range)	*N* = 847 2 (0–21)	HADS‐A (range 0–21) Median (range)	*N* = 846 3 (0–20)	–	–
Brunault et al. (2013)	HADS‐D (range 0–21) Possible depression 8–10 Probable depression ≥11	*N* = 120 12.5% 6.7%	–	–	–	–
	HADS‐D (range 0–21) Mean (*SD*)	*N* = 120 4.5 (3.6)	–	–	–	–
Chambers (2012)	BSI‐18‐D (range 0–72) Median (IQR)	*N* = 742 42 (40–50)	BSI‐18‐A (range 0–72) Median (IQR)	*N* = 742 39 (39–48)	–	–
	BSI‐18‐D T score >63	*N* = 742^b^ 6.1%	BSI‐18‐A T score >63	*N* = 742^b^ 3.4%	BSI‐18‐total T score >63	*N* = 742^b^ 4.3%
Chen et al. (2013)	UW‐QOL‐mood (range 0–100) Mean	*N* = 54 62.1	–	–	–	–
	UW‐QOL‐mood (range 0–100) Extremely depressed = 0 Somewhat depressed = 25	*N* = 54 9.0% 4.0%	–	–	–	–
Chongpison (2015)	SF‐12v2‐mental (range 100–0) Depression ≤45.6	*N* = 554 24.7%	–	–	–	–
Crespi et al. (2008)	CES‐D (range 0–60) Mean (*SD*) Range	*N* = 1,105 7.7 (7.7) 0−50	–	–	–	–
	CES‐D (range 0–60) ≥16	*N* = 1,105 13.0%			–	–
Dahl et al. (2005)	HADS‐D (range 0–21) Mean (*SD*)	*N* = 1,408 2.8 (3.1)	HADS‐A (range 0–21) Mean (*SD*)	*N* = 1,408 4.6 (3.7)		
	HADS‐D (range 0–21) ≥8	*N* = 1,408^b^ 9.7%	HADS‐A (range 0–21) ≥8	*N* = 1,408 19.2%	HADS‐A ≥8 and HADS‐D ≥8	*N* = 1,408 6.8%
Funk et al. (2012)	BDI (range 0–63) Mild 10–20 Moderate 21–30 Severe ≥31	*N* = 326^b^ 22.8% 3.3% 1.5%	–	–	–	–
Goo et al. (2016)	PHQ‐2 (range 0–2) Depressive group ≥1	*N* = 702^b^ 26.1%	–	–	–	–
Greenwald and McCorkle (2008)	CES‐D (range 0–60) ≥16	*N* = 179 47.1% 6−11 ysd (*N* = 62) 54.1% 12−15 ysd (*N* = 59) 49.1% ≥16 ysd (*N* = 68) 37.5%	–	–	–	–
Harrison et al. (2011)	HADS‐D (range 0–21) possible 8–10 probable 11–21	*N* = 633 7.3% 2.1% Breast (*N* = 254) 9.1% Colorectal (*N* = 191) 7.3% Prostate (*N* = 188) 11.7% 5−7 ysd (*N* = 372) 10.2% 9−11 ysd (*N* = 135) 7.4% 14−16 ysd (*N* = 126) 8.7%	HADS‐A (range 0–21) possible 8–10 probable 11–21	*N* = 627 13.6% 9.3% Breast (*N* = 248) 31.0% Colorectal (*N* = 190) 18.9% Prostate (*N* = 189) 15.9% 5−7 ysd (*N* = 367) 21.3% 9−11 ysd (*N* = 134) 27.6% 14−16 ysd (*N* = 126) 22.2%	–	–
Henningsohn (2002)	Visual digital scale (range 1–7); cut‐off NR	*N* = 190 38.9% 6−10 ysd (*N* = 90): 47.0% >10 ysd (*N* = 100): 32.0%	Visual digital scale (range 1–7); cut‐off NR	*N* = 191 18.8% 6−10 ysd (*N* = 92): 23.0% >10 ysd (*N* = 99): 15.0%	–	–
Hoffman et al. (2011)	–	–	–	–	K6‐scale (range 0–24) Serious psychological distress >12	*N* = 4,636 5.6%
Johansson et al. (2014)	Self‐developed questionnaire (range 0–7) Moderate/high ≥3	*N* = 339 49.0%	Self‐developed questionnaire (range 0–7) Moderate/high ≥3	*N* = 339 43.0%	–	–
McCorkle et al. (2013)	CES‐D (range 0–60) Mean Median (Range)	*N* = 202 9 6 (0–46)	–	–	–	–
	CES‐D (range 0–60) ≥16	*N* = 202 21.3%	–	–	–	–
Pedersen et al. (2019)	BDI‐II (range 0–63) moderate/severe depression >18	*N* = 309 5.4%	–	–	–	–
Reyes‐Gibby et al. (2012)	PHQ‐8 (range 0–24) Mean (*SD*) Median (range)	*N* = 240 4 (4.8) 2 (0–24)	–	–	–	–
	PHQ‐8 (range 0–24) Clinically significant depression ≥10	*N* = 240 16.2%				
Schootman et al. (2010)	–	–	–	–	K6‐scale (range 0–24) Serious psychological distress >12	*N* = 2,762 11.6%
Sharpley (2017)	SDS (range 20–80) Mean (*SD*) Range	*N* = 146 37 (9) 21−60	–	–	–	–
	SDS (range 20–80) Clinically significant depression >39	*N* = 146 39%	–	–	–	–
Vogel et al. (2017)	HADS‐D (range 0–21) Borderline/abnormal ≥8	*N* = 707 7.2%	HADS‐A (range 0–21) Borderline/abnormal ≥8	*N* = 702 18.1%	–	–

Abbreviations: BDI, Beck Depression Inventory; BSI, Brief Symptom Inventory; CES‐D, Center for Epidemiological Studies Depression questionnaire; HADS, Hospital Anxiety Depression Scale; IQR, interquartile range; K6, Kessler psychological distress scale; NR, not reported; PHQ, Patient Health Questionnaire; POMS, Profile Of Mood States; SDS, Symptom Depression Scale; SF, Short Form Health Survey; UW‐QOL, University of Washington Quality Of Life questionnaire; Ysd, years since diagnosis.

a *N* of specific measurement is given and may therefore differ from the overall number as presented in Table 1.

b Prevalence for total group of cancer survivors, not specified per tumour type.

Of the 18 studies detailing on the prevalence of depressive symptoms, the depression subscale of the Hospital Anxiety and Depression Scale (HADS‐D) was the most frequently used (*N* = 5). Other measures used were the Center for Epidemiological Studies Depression questionnaire (CES‐D), the Brief Symptom Inventory (BSI) depression subscale, the University of Washington Quality of Life questionnaire (UW‐QOL), the Short Form Health Survey (SF), the Beck Depression Inventory (BDI), the Patient Health Questionnaire (PHQ), the Symptom Depression Scale (SDS) or a self‐developed questionnaire. The reported prevalence of depressive symptoms in long‐term cancer survivors varied from 5.4% to 49.0%. The pooled prevalence of patients with depressive symptoms was estimated to be 21.0%.

A total of seven studies specifically reported on symptoms of anxiety. Most of these studies used the HADS anxiety subscale (HADS‐A) (*N* = 4). Other used measures were the BSI anxiety subscale or a self‐developed questionnaire. The reported prevalence of anxiety ranged from 3.4% to 43.0%. The pooled prevalence of patients with symptoms of anxiety was estimated to be 21.0%.

In total, four studies reported on distress. Most studies used the Kessler Psychological Distress Scale (K6‐scale) to measure symptoms of distress (*N* = 2). Other measures used were the HADS total score and the BSI total score. The prevalence of reported distress ranged from 4.3% to 11.6%. The mean reported percentage of patients with distress symptoms was estimated to be 7.0%.

**Figure 2:**
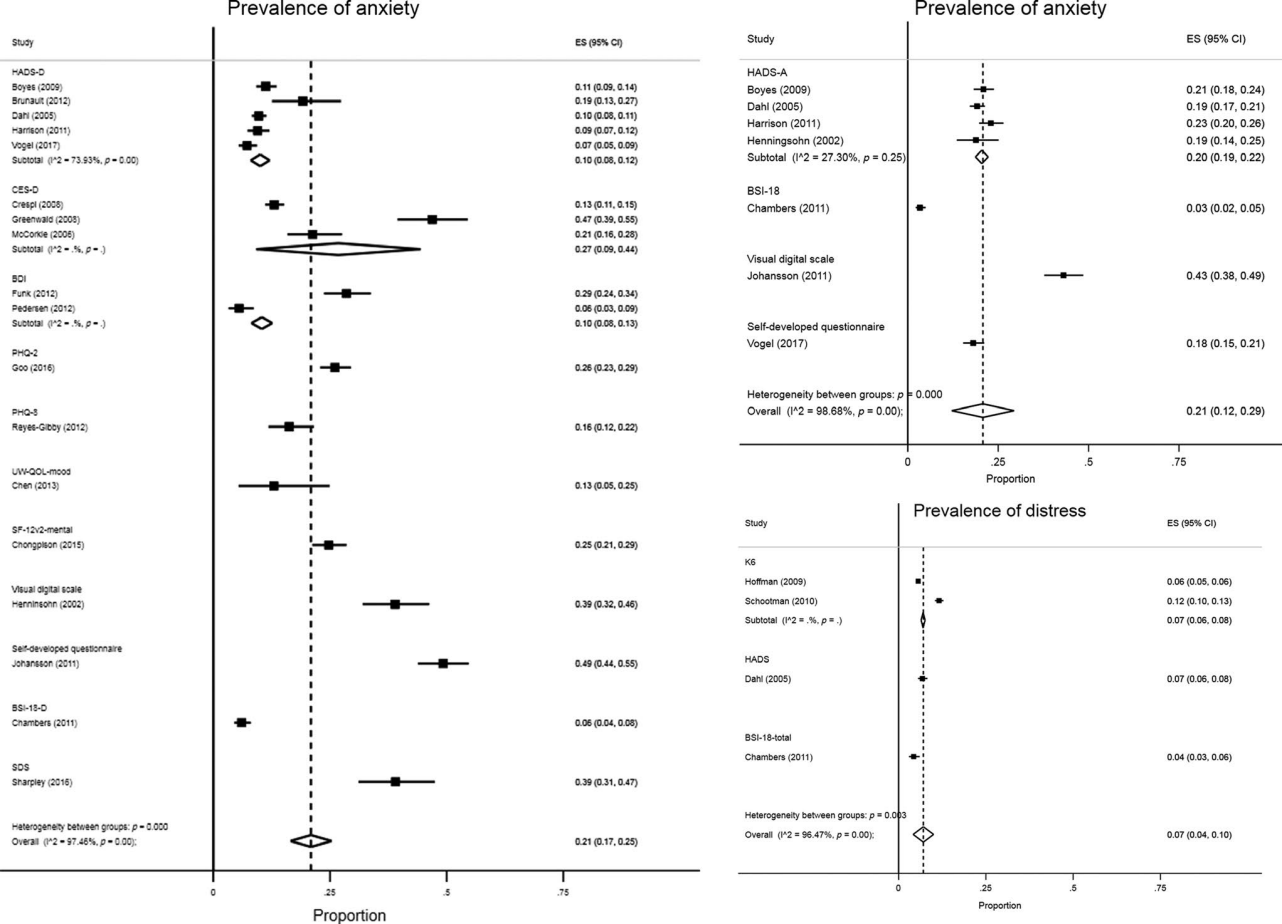
Forest plots of prevalence of symptoms of depression, anxiety and distress

Further details in regard to symptoms of depression, anxiety and distress, and the created forest plots can be found in Table 2 and Figure 2 respectively.

### 3.3 Quality assessment

**Figure 3:**
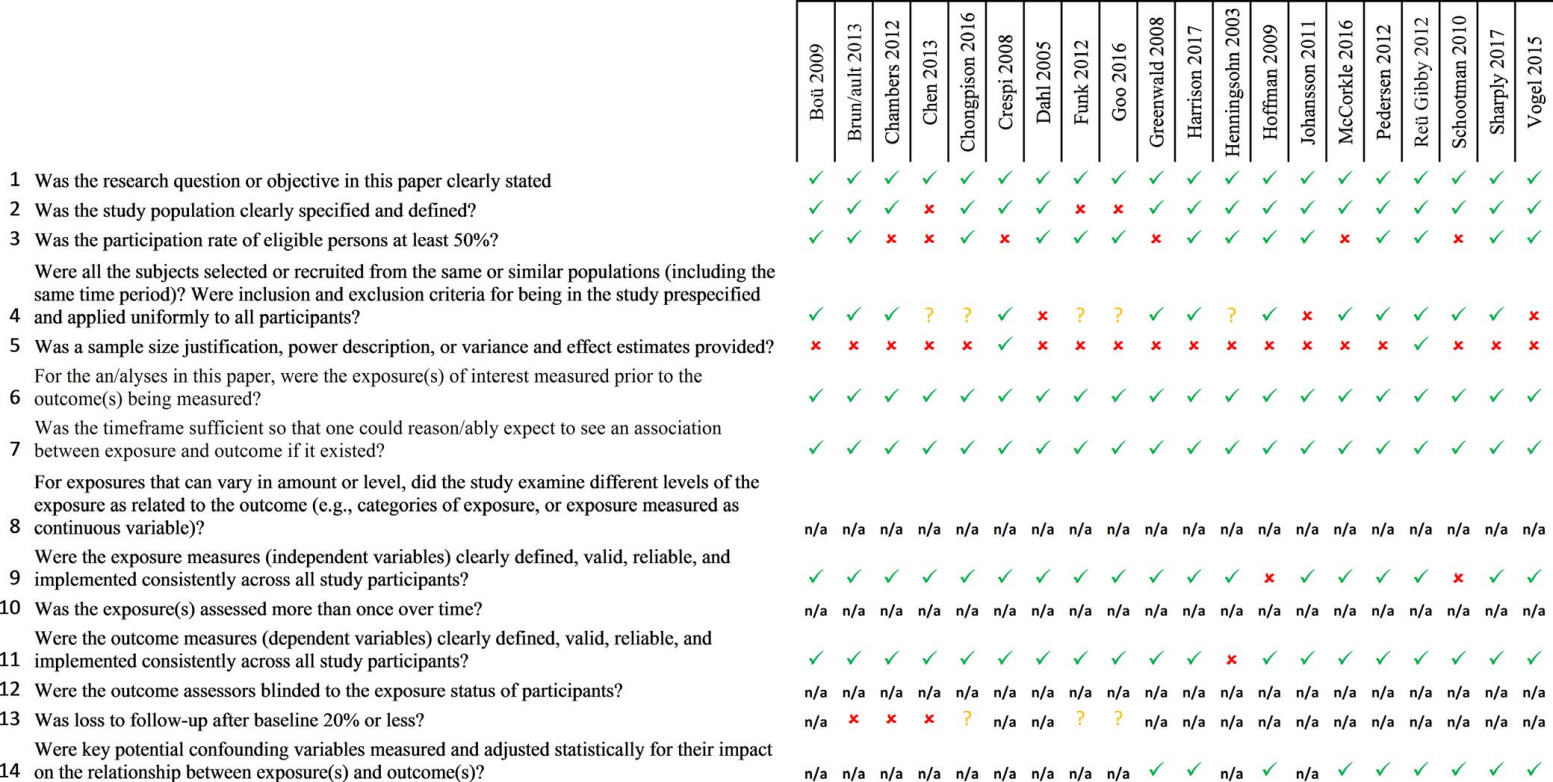
Risk of bias

The summary scores of the quality assessment were “excellent” for half of the included studies (*N* = 9), nine of the included studies were of “good” quality and two studies were scored as having “fair” quality. The shortcomings mostly identified were (a) a participation rate of <50% (*N* = 6) and (b) lack of clarity in the description of the study population (*N* = 3). See Figure 3 for more details.

## 4 DISCUSSION

### 4.1 Main findings

In this systematic review, we reported on the prevalence of symptoms of depression, anxiety and distress in cancer survivors, five or more years after diagnosis. The pooled prevalence of symptoms of depression and anxiety was 21.0%; the pooled prevalence of distress was 7.0%. Most frequently used instruments to measure these psychological symptoms were the HADS and the CES‐D.

### 4.2 Interpretation of the findings

Our results suggest that the prevalence of symptoms of depression, anxiety and distress among long‐term survivors is comparable with, or even slightly below, the prevalence of these symptoms in the general population.

Looking more specifically at symptoms of depression, measured with the HADS‐D, prevalence rates in general and elderly populations between 10.0% and 23.0% have previously been reported (Djukanovic, Carlsson, & Årestedt, 2017; Hinz & Brahler, 2011). In our study, in long‐term cancer survivors, the pooled prevalence of symptoms of depression, measured with this same questionnaire, was quite low, that is, only 10.0% (8.0%–13.0%). Measurement of symptoms of depression in the general population, using the CES‐D, showed a prevalence of 21.0% (Smarr & Keefer, 2011), which is a bit lower compared to the 27.0% (9.0%–44.0%) in our study. The prevalence of symptoms of anxiety in the general population, according to the HADS‐A, has been found to be between 10.0% and 21.0% (Djukanovic et al., 2017; Hinz & Brahler, 2011). The pooled prevalence of 20.0% (19.0%–22.0%) found in our study in cancer survivors is on the high end of this range.

These relatively low prevalence rates in long‐term cancer survivors are in contrast to prevalence rates found among cancer patients during and shortly after diagnosis and treatment, when higher prevalence rates of psychological symptoms are found (Maass et al., 2015; Mitchell, Ferguson, Gill, Paul, & Symonds, 2013; Watts et al., 2015). Three aspects might explain this difference: (a) former studies did not focus specifically on the period beyond the first 5 years after cancer diagnosis, as we did. Earlier research showed that adults who have survived cancer for at least 5 years frequently identify themselves as cancer survivors and/or as ex‐patients, rather than as victims or patients (Deimling, Bowman, & Wagner, 2007). So, the longer time since diagnosis, and potential regained trust in one's health, might positively influence psychological functioning of these survivors; (b) cancer patients who are suffering from symptoms of depression, anxiety and/or distress might be less inclined to participate or continue participation in studies regarding psychological sequelae, and herewith be missing in our prevalence data. In line with this, an earlier meta‐analysis showed that higher levels of depressive symptoms predict higher mortality rates (Pinquart & Duberstein, 2010). It is therefore possible that depressed patients are less frequently long‐term cancer survivors and as a result were not included in studies in our review; and (c) cancer patients dealing with depressive, anxious and/or distress symptoms, early after their diagnosis, could have received psychological treatment, limiting their symptoms on the long term. However, we have no data in our study on possible psychological interventions or therapies received by the patients, suffering from psychological symptoms ≤5 years after diagnosis.

Some noteworthy heterogeneity issues in reported prevalence rates were observed in this systematic review. That is, in the study of Johansson et al. (2011), among survivors of prostate cancer, higher prevalence rates of symptoms of depression (49.0%) and anxiety (43.0%) were reported. However, a self‐developed questionnaire was used, consisting of visual analogue scales for both symptoms of depression and anxiety, in which the highest five out of seven categories indicated symptoms of depression and anxiety. This relatively low cut‐off could explain the high prevalence in this study (Johansson et al., 2011). The same applies to the study of Sharpely et al (2017), in which a high prevalence of symptoms of depression (39.0%) was reported as well (Sharpley et al., 2017). Yet, they used the Zung Self‐rating Depression Scale, with a cut‐off of ≥40 (range 20–80), known to lead to high numbers of false‐positive classifications (Yesavage et al., 1982). On the other hand, in the study of Chambers et al. (2012), among survivors of colorectal cancer, a very low (6.0%) prevalence of symptoms of depression was reported (Chambers et al., 2012). The prevalence rate in this study was measured among survivors in a longitudinal study, of whom about 40.0% were enrolled 60 months after diagnosis. Therefore, the low prevalence could potentially be explained by healthy survivor bias.

Studies using the HADS depression and anxiety scales showed a high agreement in our review. However, there was some variability among studies using the CES‐D. Interestingly, the difference among studies using the CES‐D was largest for two specific studies, apparently reporting on the same patient population (Greenwald & McCorkle, 2008; McCorkle et al., 2006). We were unable to hypothesise an explanation for the difference in these studies, except that there was an age difference in the samples. The slightly older population in the study of McCorkle et al. (2006) reported a lower prevalence though, which is contradictory to most literature suggesting an increased prevalence of depressive symptoms with increasing age (Djukanovic et al., 2017). In order to enhance comparability and interpretation in future research into prevalence rates, we suggest using validated and widely used measures to enhance comparability and interpretation.

### 4.3 Strengths and weaknesses

A major strength of our review is that we only included studies that presented data on the prevalence of symptoms of depression, anxiety and distress in cancer survivors, five or more years after their cancer diagnosis. This enabled us to provide valuable new insights into these symptoms, explicitly in long‐term cancer survivors, most of whom are no longer being followed in routine hospital follow‐up. Focusing merely on symptoms and excluding clinical diagnoses of depression and anxiety disorders is another strength of our study. It gives a more accurate estimate of the symptoms’ prevalences, which is important since clinically diagnosed patients often have specific healthcare needs and treatments. Furthermore, focusing on symptoms only is essential in case of early detection and early intervention programmes. Finally, our study presented pooled prevalences of symptoms of depression, anxiety and distress, using different questionnaires, enabling future comparisons.

Nevertheless, we also identified several weaknesses in our systematic review. First, since we included studies describing a variety of cancer types, we presented data on a heterogeneous population of cancer survivors. This hampers description of the prevalence for specific patients’ groups. Due to the amount of studies found among long‐term survivors, and because a fair proportion of included studies report on populations with multiple cancer types, subgroup analyses for cancer types were not possible. Second, most of the studies we included were not designed to study the prevalence of symptoms of depression, anxiety and/or distress. Rather, these were mostly secondary outcomes in studies, designed to assess relationships between exposures and outcomes among cancer patients. Yet, due to the total number of patients included (*N* = 17,726), we believe our results to hold quite some value. Lastly, the quality assessment tool used in the current study was not specifically designed for prevalence studies. However, the items used to assess overall quality were adapted to our review question.

### 4.4 Implications for (primary) care and conclusion

Our results suggest that the prevalence rates of symptoms of depression, anxiety and distress among long‐term cancer survivors do not fundamentally differ from the general population. Most hospitals have follow‐up schedules for patients after the treatment for cancer, lasting up to 5 years after treatment. Hereafter, in healthcare systems with a gatekeeping function for GPs, these physicians function as the primary physician for long‐term cancer survivors. Earlier research showed that patients with cancer frequently consult their primary care physician for psychosocial issues, starting in the first years after diagnosis (Brandenbarg et al., 2017; Roorda, Berendsen, Groenhof, van der Meer, & de Bock, 2013). Apart from providing or referring to psychological care at the short term, GPs might reassure cancer survivors that psychosocial sequelae are most prevalent in the first years and likely to decline over time. Based on our findings, there seems to be no need for primary care physician or other (primary) healthcare providers to actively screen all long‐term cancer survivors for symptoms of depression, anxiety and/or distress.

## CONFLICT OF INTEREST

No conflicts to declare.

## APPENDIX 1

### Search string

(((((((cancer*[ti] OR neoplasm*[ti] OR oncol*[ti]))) AND ((surviv*[ti] OR long term*[ti] OR long‐term*[ti] OR longterm*[ti]))) AND (("Depression"[Mesh] OR "Anxiety"[Mesh] OR depress*[tiab] OR dysthym*[tiab] OR anxiety*[tiab] OR distress*[tiab])))) AND ("Prevalence"[Mesh] OR prevalen*[tiab])) NOT (((((((((cancer*[ti] OR neoplasm*[ti] OR oncol*[ti]))) AND ((surviv*[ti] OR long term*[ti] OR long‐term*[ti] OR longterm*[ti]))) AND (("Depression"[Mesh] OR "Anxiety"[Mesh] OR depress*[tiab] OR dysthym*[tiab] OR anxiety*[tiab] OR distress*[tiab])))) AND ("Prevalence"[Mesh] OR prevalen*[tiab])) AND ((infant[MeSH] OR child[MeSH] OR adolescent[MeSH]))) NOT ((((((((cancer*[ti] OR neoplasm*[ti] OR oncol*[ti]))) AND ((surviv*[ti] OR long term*[ti] OR long‐term*[ti] OR longterm*[ti]))) AND (("Depression"[Mesh] OR "Anxiety"[Mesh] OR depress*[tiab] OR dysthym*[tiab] OR anxiety*[tiab] OR distress*[tiab])))) AND ("Prevalence"[Mesh] OR prevalen*[tiab])) AND (adult[MeSH]))) Filters: Publication date from 2000/01/01.
